# Examining safety of cardiac surgery in patients with preoperative cardiac arrest

**DOI:** 10.1371/journal.pone.0319563

**Published:** 2025-03-11

**Authors:** Amulya Vadlakonda, Syed Shahyan Bakhtiyar, Shayan Ebrahimian, Sara Sakowitz, Nikhil Chervu, Arjun Verma, Corynn Branche, Khajack Darbinian, Peyman Benharash

**Affiliations:** 1 Department of Surgery, Center for Advanced Surgical and Interventional Technology, University of California, Los Angeles, California, Unites States of America,; 2 Department of Surgery, University of California, Los Angeles, California, Unites States of America,; 3 Department of Surgery, University of Colorado, Aurora, Colorado, Unites States of America,; 4 Division of Cardiac Surgery, Department of Surgery, University of California, Los Angeles, California, Unites States of America; Ataturk University Faculty of Medicine, TÜRKIYE

## Abstract

**Background:**

Although postoperative cardiac arrest is a well-studied complication of cardiac surgery, few guidelines exist regarding timing of surgery in preoperative cardiac arrest (pCA). We examined the association between delayed timing of operation and postoperative outcomes following cardiac surgery in a large cohort of pCA.

**Methods:**

Adults with a diagnosis of pCA undergoing a cardiac operation were identified in the 2016-2020 National Inpatient Sample. Those requiring surgery within 24 hours fo cardiac arrest were excluded. Patients who underwent a cardiac procedure after 5 days of cardiopulmonary resuscitation were classified as *Delayed* (others: *Early*). Multivariable regression models were constructed to evaluate associations between delayed timing of surgery with in-hospital mortality, postoperative complications, hospitalization duration, and costs.

**Results:**

Of an estimated 9,240 patients meeting study criteria, 4,860 (52.6%) received delayed cardiac surgery. Following entropy balancing, delayed surgery was significantly associated with decreased odds of in-hospital mortality (Adjusted Odds Ratio [AOR] 0.75, 95% Confidence Interval [CI] 0.58 – 0.97). However, delayed operation demonstrated greater odds of postoperative thromboembolic (AOR 1.44, 95% CI 1.02 – 2.04), and infectious (AOR 1.65, 95% CI 1.31 – 2.08) complications. Notably, delay did not alter odds of neurologic complication, and was linked to a decrement in per-day costs (β -$2,100, 95% CI -2,600 – −1,700).

**Conclusions:**

While preoperative cardiac arrest remains challenging, the present study demonstrates the safety profile of delaying cardiac operation among patients tolerating at least 24 hours of a delay to surgery. Future studies are needed to elucidate the factors associated with favorable outcomes in this population.

## Introduction

The decision to operate on patients who have sustained a cardiac arrest is often complex and creates clinical as well as ethical dilemmas for the surgical team. Although highly variable, patients with preoperative cardiac arrest (pCA) may suffer from end organ injury and importantly, neurologic insult. In many instances, however, surgery is required to address the anatomic root cause of cardiac arrest and offer a chance for recovery.

Even in the contemporary era, cardiac arrest continues to portend a significant risk of mortality. Cardiac surgery under such circumstances is particularly risky and while lifesaving, may result in serious cerebral, renal, and pulmonary dysfunction, among others. Thus, practitioners may elect to delay surgery until adequate organ recovery is achieved. With scarcity of literature available regarding the optimal timing of surgical intervention following cardiac arrest, deductions may be drawn from work done on myocardial infarction. Consensus guidelines for ST-elevation myocardial infarction recommend intervention as soon as clinically feasible to maximize the chance for neurologically intact survival [1]. Unlike in ST-elevation myocardial infarction, however, studies examining the optimal time from pCA to cardiac surgery are lacking. Furthermore, increasing emphasis on surgical quality improvement and public reporting programs may, in fact, discourage surgeons to undertake high-risk operations [2–4], such as those in patients with cardiac arrest.

In the present work, we characterized the clinical and financial outcomes of early and delayed cardiac surgery following pCA using a large, contemporary cohort. We hypothesized that delaying surgery would be safe. We further explored whether a delayed operation would be linked to increased postoperative complications and costs.

## Materials and Methods

### Data source

This was a retrospective cohort study using the 2016-2020 National Inpatient Sample (NIS). The NIS is the largest publicly available all-payer inpatient database in the US. Maintained by the Agency for Healthcare Research and Quality as a part of the Healthcare Cost and Utilization Project (HCUP), the NIS samples 20% of all discharges from participating centers. Using survey-weighting methodology, this database provides accurate estimates for approximately 97% of all US hospitalizations. All results are reported with survey weights applied.

### Study population, variables, and outcomes

**Fig 1:**
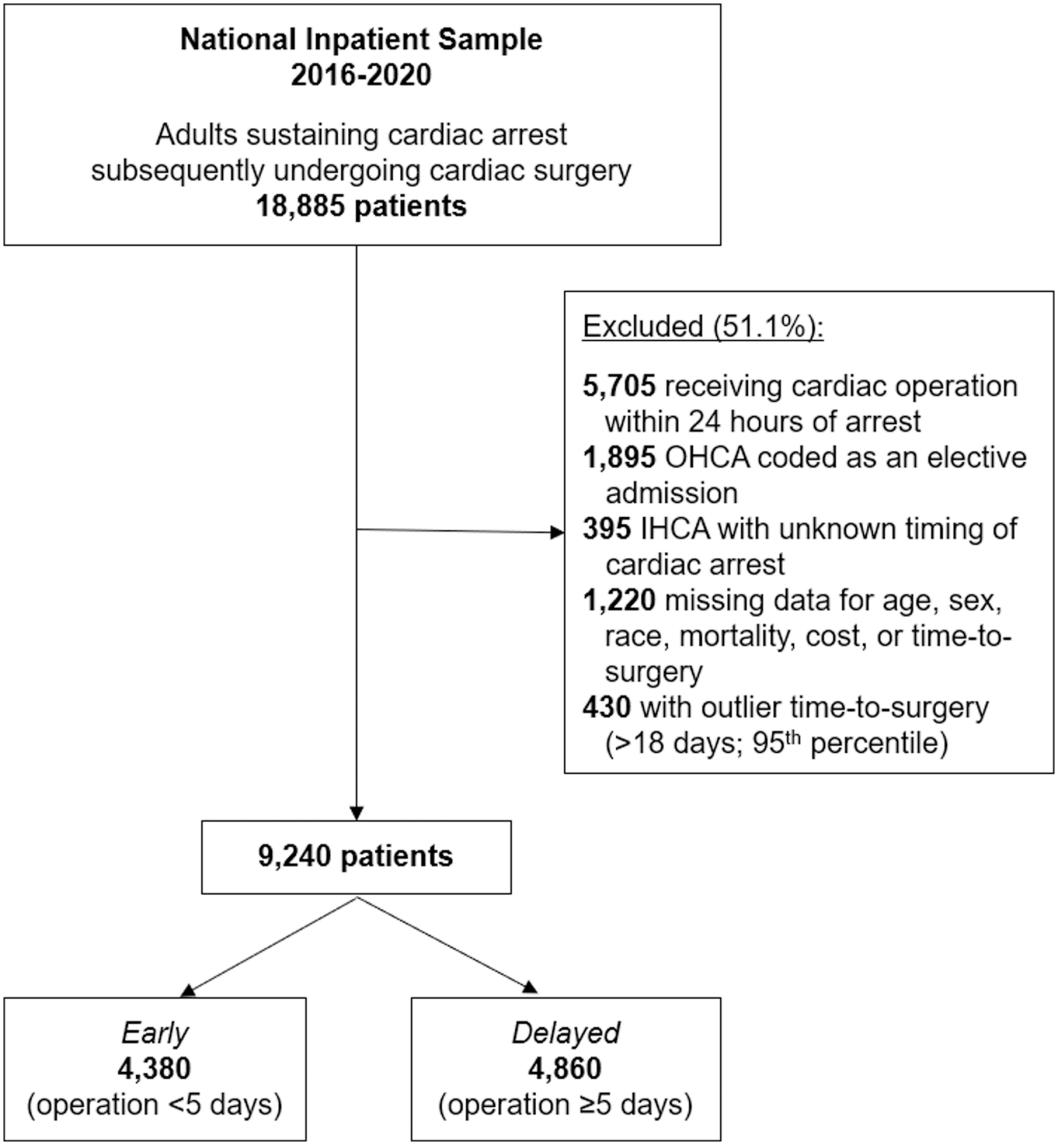
Study CONSORT diagram. ECMO, extracorporeal membrane oxygenation; IHCA, in-hospital cardiac arrest; OHCA, out-of-hospital cardiac arrest.

**Fig 2:**
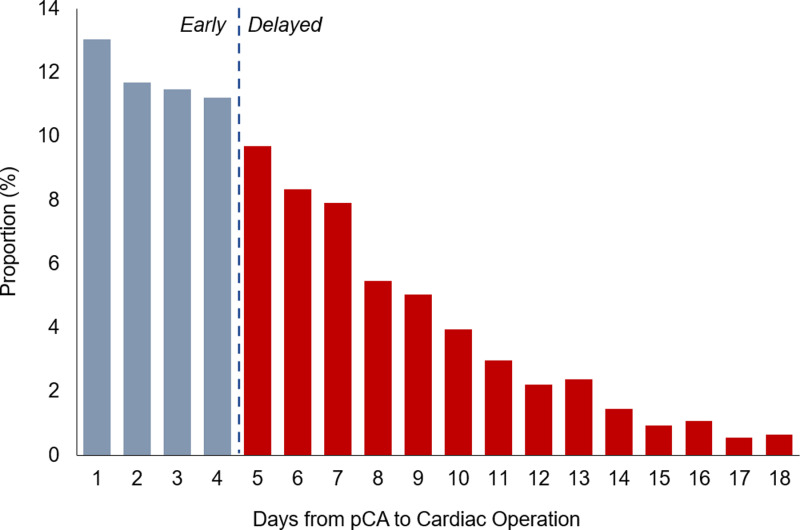
Distribution of time to cardiac surgery following preoperative cardiac arrest (pCA). Patients were considered to have a delayed operation after the median of 5 days from the time of arrest.

All adult (≥18 years) admissions with a diagnosis of cardiac arrest were tabulated using *International Classification of Disease, Tenth Revision* (ICD-10) codes that have been extensively validated in previous studies [5–8]. To reduce selection bias, only patients who subsequently underwent cardiac surgery and tolerated at least a 24-hour delay to operation were included (Fig 1). Using previously reported methodology, the cohort was further stratified into out-of-hospital (OHCA) and preoperative in-hospital cardiac arrests (IHCA). Time-to-surgery following cardiac arrest was ascertained using the procedure day variable (“PRDAY”) provided by NIS. All OHCA patients were considered to have preoperative arrest, while IHCA on the same day or after the index operation, were excluded. To mitigate the effects of outliers, the top 5^th^ percentile of time-to-surgery were excluded from analysis. Lastly, records with missing age, race, mortality, or cost data were similarly excluded. Patients who underwent cardiac surgery 5 days (median time for the cohort) following cardiac arrest comprised the *Delayed* group; all others were considered *Early* (Fig 2).

Relevant patient and hospital characteristics, including age, sex, race, income quartile, insurance status, and hospital teaching status, were defined in accordance with the NIS Data Dictionary. Individual patient diagnoses and procedures were identified using ICD-10 codes. The modified Elixhauser Comorbidity Index, a previously validated composite of 30 chronic conditions, was used to quantify the burden of chronic illness [9]. The primary outcome of the study was in-hospital mortality, while secondary outcomes included postoperative complications, postoperative length of stay (pLOS), and hospitalization costs. Postoperative complications were grouped as thromboembolic, respiratory, infectious, renal, or neurological. Hospitalization charges were calculated by application of hospital-specific cost-to-charge ratios to overall hospitalization costs followed by inflation adjustment to the 2020 Personal Health Care Price Index [10]. Costs were subsequently normalized to length of stay to calculate per-day costs, reported in USD.

### Statistical analysis

The Pearson’s χ^2^, adjusted Wald, and Mann Whitney U-tests were employed for bivariate comparisons of patient and hospital characteristics. To assess the significance of temporal trends across the study period, we used Cuzick’s non-parametric test across ordered groups (nptrend) [11]. Entropy balancing was applied to adjust for intergroup differences between *Early* and *Delayed* cohorts. Found to be superior to propensity score matching in observational studies, this method uses an entropy reweighting methodology to balance covariate distributions, while retaining the entire sample for analysis [12]. Entropy balancing has been demonstrated as a robust method to account for differences in baseline characteristics between comparative groups [13,14]. Multivariable logistic and linear regression models were developed to evaluate the risk-adjusted associations of delayed timing of surgery with outcomes of interest. To reduce bias, model covariates were selected by the Least Absolute Shrinkage and Selection Operator (LASSO). This methodology reduces overfitting and increases out-of-sample reliability [15]. Final models adjusted for age, sex, income quartile, payer status, patient comorbidities, MCS initiation, operation type, and year of admission.

Categorical variables are reported as proportions, while continuous variables are reported as means with standard distributions or medians with interquartile ranges, as appropriate. Models were evaluated and optimized using receiver-operating characteristics (c-statistic) as well as Akaike and Bayesian information criteria. Logistic and linear regression outputs are reported as adjusted odds ratios (AOR) and β coefficients, respectively, both with 95% confidence intervals (CI). Stata 16.0 software (StataCorp LP, College Station, TX) was used for statistical analysis. This study was deemed exempt from full review by the Institutional Review Board at the University of California, Los Angeles.

## Results

**Fig 3:**
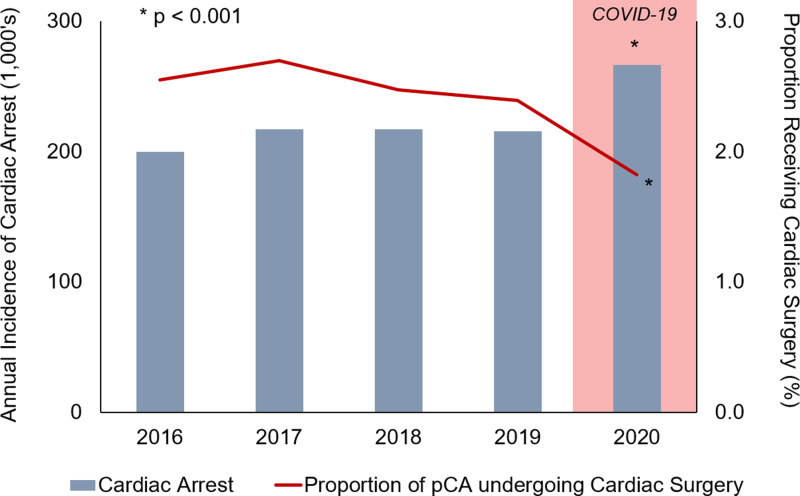
Annual incidence of cardiac arrest and proportion undergoing subsequent cardiac surgery. pCA, preoperative cardiac arrest.

Of an estimated 9,240 patients who had pCA and subsequent cardiac surgery from 2016-2020, 4,860 (52.6%) were *Delayed*. Although the annual incidence of cardiac arrest hospitalizations increased significantly during the study period (p < 0.001), the proportion of patients who receive a subsequent cardiac operation after pCA declined, with a nadir in 2020 (Fig 3).

The full comparison of patient and hospital demographics is shown in Table 1. While both groups had a similar distribution of age and sex, *Delayed* had a greater burden of comorbidities (4.7 ±  1.5 vs 4.4 ±  1.6, p < 0.001). Specifically, *Delayed* more frequently had congestive heart failure (67.2 vs 54.1%, p < 0.001), neurologic conditions (27.1 vs 20.8%, p = 0.002), and end-stage renal disease (11.3 vs 6.6%, p < 0.001). Compared to others, *Delayed* were more commonly Black (12.4 vs 7.6%) and Hispanic (9.3 vs 7.9%, both p = 0.001), insured by Medicaid (12.4 vs 7.9%, p = 0.001), and in the lowest quartile of income (30.5 vs 27.6%, p = 0.009). While CABG was the most common operation among both cohorts, *Delayed* was more likely to undergo both isolated (22.5 vs 16.9%) and multi-valve procedures (4.8 vs 2.2%, both p < 0.001).

**Table 1:** Demographic, clinical, and hospital characteristics. *CABG, coronary artery bypass graft; MCS, mechanical circulatory support (intra-aortic balloon pump or percutaneous ventricular assist device).*

	*Early* (n = 4,380)	*Delayed* (n = 4,860)	*P-value*
Age, years	65.6 ± 11.8	64.6 ± 13.2	0.09
Female, %	28.1	29.0	0.66
Elixhauser Comorbidity Index	4.4 ± 1.6	4.7 ± 1.5	<0.001
**Race, %**			0.001
White	75.3	72.3	
Black	7.6	12.4	
Hispanic	7.9	9.3	
Asian/Pacific Islander	3.9	2.7	
Other ^a^	5.3	3.3	
**Income Quartile, %**			0.009
76–100^th^	22.8	19.3	
51–75^th^	25.5	21.2	
26–50^th^	24.1	29.0	
0–25^th^	27.6	30.5	
**Payer, %**			0.001
Private	30.0	23.8	
Medicare	54.2	55.9	
Medicaid	7.9	12.4	
Other ^b^	7.9	7.9	
**Comorbidities, %**			
Congestive heart failure	54.1	67.2	<0.001
Coronary artery disease	51.8	46.8	0.03
Hypertension	62.6	59.7	0.21
Pulmonary hypertension	6.6	10.3	0.006
Neurologic condition	20.8	27.1	0.002
Diabetes	29.5	25.1	0.04
Liver disease	12.8	14.0	0.44
Coagulopathy	23.4	20.4	0.11
End-stage renal disease	6.6	11.3	<0.001
**Operation Type, %**			<0.001
Isolated CABG	69.2	60.7	
Isolated Valve	16.9	22.5	
CABG ^+^ Valve	11.6	11.9	
Multi-Valve	2.2	4.8	
**MCS Initiation, %**			<0.001
None	66.6	77.3	
Preoperative	17.0	8.6	
At time of operation	12.9	10.7	
Postoperative	3.5	3.4	
**Hospital Type, %**			0.60
Metropolitan teaching	83.1	84.5	
Metropolitan non-teaching	15.0	13.5	
Non-metropolitan	1.9	2.0	
**Hospital Size, %**			0.01
Large	61.2	67.4	
Medium	27.4	24.2	
Small	11.4	8.4	

^a^Combined group of other races, defined by NIS.

^b^Combined group of self-pay, uninsured, and other insurance statuses, defined by NIS.

On unadjusted comparison, both groups had similar rates of in-hospital mortality (17.1 vs 20.2%, p = 0.08). However, the *Delayed* cohort more frequently experienced thromboembolic (4.8 vs 1.6%, p < 0.001) and infectious (19.7 vs 13.1%, p < 0.001) complications. The most common complication in both groups was acute kidney injury (44.7 vs 43.0%, p = 0.49). There was no difference between groups for neurologic and respiratory complications (Table 2). Although pLOS was similar between both groups (p = 0.13), the *Delayed* patients had lower per-day costs compared to *Early* counterparts (USD $4,800 [3,800 – 6,500] vs $6,400 [4,700 – 8,900], p < 0.001). To examine the possible effect of the COVID-19 pandemic, a subgroup analysis conducted among only admissions in 2020 demonstrated acceptably similar outcomes (Table 2).

**Table 2:** Unadjusted outcomes for delayed cardiac surgery, relative to early operation. *LOS, length of stay; USD, United States dollars.*

		*2016-2020*		*2020 only (COVID-19)*		
	*Early*	*Delayed*	*P-value*	*Early*	*Delayed*	*P-value*
**Clinical outcomes, %**						
In-hospital mortality	20.2	17.1	0.08	17.8	14.9	0.43
Neurological complication	2.9	2.3	0.40	3.4	1.0	0.12
Thromboembolic complication	1.6	4.8	<0.001	2.8	5.6	0.19
Respiratory complication	32.6	36.5	0.08	29.6	33.3	0.42
Infectious complication	13.1	19.7	<0.001	12.8	22.1	0.03
Acute kidney injury	43.0	44.7	0.49	47.5	43.6	0.46
Requirement for transfusion	21.7	21.5	0.92	17.9	22.1	0.31
**Resource utilization**						
Postoperative LOS (days)	8 [5–15]	8 [5–16]	0.13	8 [5–16]	8 [5–15]	0.38
Cost (USD $1,000)	6.4 [4.7–8.9]	4.8 [3.8–6.5]	<0.001	6.7 [4.9–9.0]	5.0 [4.0–7.0]	<0.001

**Fig 4:**
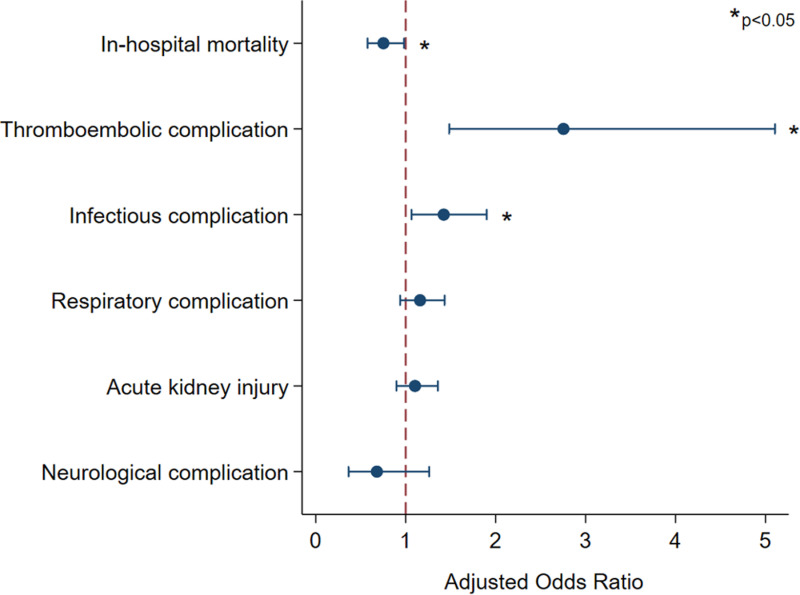
Adjusted outcomes following cardiac surgery after preoperative cardiac arrest.

As demonstrated by minimal standard mean differences in covariates (S1 Figure), adequate entropy balancing was achieved. Following entropy balancing and multivariable adjustment, several key associations were observed (Fig 4). Notably, delayed surgery was associated with lower odds of in-hospital mortality (AOR 0.75, 95% CI 0.58 – 0.97). Nonetheless, delayed operation was linked to greater odds of thromboembolic (AOR 2.59, 95% CI 1.39 – 4.80) and infectious complications (AOR 1.48, 95% CI 1.12 – 1.96). Importantly, there was no association between delayed cardiac surgery and neurologic complications (AOR 0.72, 95% CI 0.40 – 1.31). While delayed operation was not associated with a significant increment in pLOS (β + 0.47, 95% CI -0.90 – 1.83), it remained linked to decreased per-day costs (β -$2,100, 95% CI -2,600 – -1,700). Complete adjusted outcomes are shown in Table 3.

**Table 3:** Adjusted outcomes for delayed cardiac surgery, relative to early operation.

	*Delayed*	*95% Confidence Interval*	*P-value*
**Clinical outcomes, %**			
In-hospital mortality	0.75	0.58 – 0.97	0.03
Neurological complication	0.72	0.40 – 1.31	0.28
Thromboembolic complication	2.59	1.39 – 4.80	0.003
Respiratory complication	1.17	0.95 – 1.43	0.14
Infectious complication	1.48	1.12 – 1.96	0.006
Acute kidney injury	1.13	0.92 – 1.39	0.23
Requirement for transfusion	0.92	0.93 – 1.16	0.48
**Resource utilization**			
Postoperative LOS (days)	0.47	− 0.90 – 1.83	0.50
Cost (USD $1,000)	−2.1	−2.6 – -1.7	<0.001

*CI, confidence interval; *LOS,* length of stay; *USD*, United States dollars.

## Discussion

The present study analyzed a national cohort of adults undergoing cardiac operations at least 24h following pCA. Although significant in clinical practice, there is a paucity of available literature on the appropriate timing of cardiac surgery after arrest. In the absence of societal or consensus guidelines, our study notes a median delay of 5 days to surgery and demonstrates the overall safety of such delays among patients sustaining cardiac arrest. Several of these findings warrant further discussion.

Cardiac arrest can be caused by a myriad of processes which include coronary artery disease, structural heart disease, arrhythmias, drug intoxication, and pulmonary embolism, among others [16–22]. Etiology aside, cardiac arrest victims are at risk for hypotension, recurrent arrest, and end-organ injury, even after return of spontaneous circulation (ROSC) [23–25]. In a multicenter study of ~ 9000 patients, James and colleagues reported hypotension in approximately 47% of patients with ROSC following a cardiac arrest, with 95% of those patients having evidence of hypotension over the first 24 hours post-arrest [26]. The cause of this hypotension is multifactorial and could be an amalgamation of several underlying derangements, such as global myocardial stunning, acute coronary syndrome, adrenal insufficiency, impaired autonomic regulation, and systemic inflammatory responses [27,28]. As such, the treatment of cardiac arrest extends beyond achieving ROSC, while optimal post-resuscitation care remains crucial to ultimate survival [29,30].

Following arrest, a subset of patients ultimately requires operative intervention to correct the underlying anatomic pathology. In the present study, we found the proportion of patients undergoing cardiac surgery to have decreased over time, with a significant drop by 2020. This may, in fact, represent the effect of the COVID-19 pandemic, which may have exacerbated existing trends. Retrospective analyses have found institutional cardiac surgery volume to have decreased by over 50% in the acute months of the pandemic [31]. Patients requiring urgent intervention may have been further impacted by lower available resources and understaffing, which may have increased delays to surgery. Importantly, despite such a decline, the incidence of cardiac arrest continues to rise, underscoring the need for clarity regarding optimal post-resuscitation surgical practices.

Our study demonstrated a significant risk of death among patients undergoing an operation after cardiac arrest, with > 20% of patients experiencing in-hospital mortality. Nonetheless, among those able to tolerate at least 24h of delay to surgery, a late operation was associated with reduced odds of death, suggesting such delay to not only be safe in this population, but may even be linked to a survival benefit. When a cardiac operation is indicated, it remains unknown whether immediate intervention would offer a greater chance at postoperative recovery, or whether a delay would improve preoperative medical optimization. In the case of myocardial infarction, for example, the American College of Cardiology and American Heart Association Taskforce suggests delaying elective surgery for 4-6 weeks after a myocardial infarction [32]. Similarly, in a study of over half a million patients undergoing non-cardiac surgery, Livhits et al. reported a significantly elevated risk of postoperative mortality in patients who underwent surgery within 60 days of a myocardial infarction [33]. However, these reports involved patients undergoing non-cardiac operations. This idea is supported by the findings of Weiss et al. who found significantly lower mortality rates in patients who under a myocardial infarction and subsequently underwent coronary artery bypass grafting 3 or more days after the primary insult [34]. Taken together, our findings are bolstered by prior work in myocardial infarction demonstrating delayed operation to potentially represent a cohort of healthier or more recovered patients. At worst, delayed surgery after cardiac arrest appears to be a safe option; at best, our findings indicate a potential survival benefit associated with a delayed operation when the etiology of cardiac arrest is unknown. Although individual circumstances and clinical picture undoubtedly determine the optimal timing for surgery, further studies may to elucidate whether operative delay achieves meaningful end-organ recovery.

In the present work, patients who underwent delayed cardiac surgical intervention had greater odds of thromboembolic and infectious complications. While patients with delayed surgery had greater comorbidities, which may predispose the development of perioperative complications, such a finding more likely reflects the iatrogenic sequelae inherent to longer hospitalizations [35]. While our analysis revealed no difference in postoperative length of stay between groups, the preoperative delay of at least 5 days may pose a clinically significant thrombogenic or infectious risk [36]. That said, late surgery did not alter odds of respiratory, renal, or neurologic complications, suggesting the risk of end-organ damage not to be elevated in the setting of a delayed operation. Given the overall safety profile of delayed surgery observed in the present study, per-day costs were in fact lower among those with a late operation. Thus, our findings additionally support the overall economic feasibility of what appears to be a clinically beneficial delay in cardiac surgery post-arrest.

The present study has several important limitations inherent to its retrospective study design. Because the NIS is an administrative database and thus lacks granularity in clinical data, we were unable to adjust for time and location of pCA, time to ROSC, laboratory values, and need for prolonged mechanical ventilation. These factors determine the timing of cardiopulmonary resuscitation and have been implicated in post-arrest survival. Furthermore, we are limited by the granularity of the ICD codes in the determination of etiology of cardiac arrest. We acknowledge the presence of potential survival bias with delayed patients representing a cohort that is more likely to survive. Thus, we excluded patients receiving an operation within 24 hours of admission to reduce cohort heterogeneity. We further used doubly robust risk adjustment and careful consideration of model covariates to reduce bias.

## Conclusions

While the decision to operate on patients recovering from cardiac arrest remains clinically complex, we demonstrate the overall safety profile and economic feasibility of delaying cardiac surgery for at least 5 days after cardiac arrest. To mitigate survival bias, the analysis was limited to patients able to tolerate at least 24h of delay. Future work is needed to expound upon the mechanisms and potential prognostic factors that may be associated with favorable outcomes following cardiac surgery among post-arrest patients.

## Supporting Information

S1 Figure Balance plot demonstrating adequate covariate entropy balancing using representative standard mean differences. (TIF)
