# Supplementary figures and images for: Examining safety of cardiac surgery in patients with preoperative cardiac arrest
Source: PLoS One. 2025 Mar 11;20(3):e0319563. doi: 10.1371/journal.pone.0319563 (PMC11896030; doi:10.1371/journal.pone.0319563)

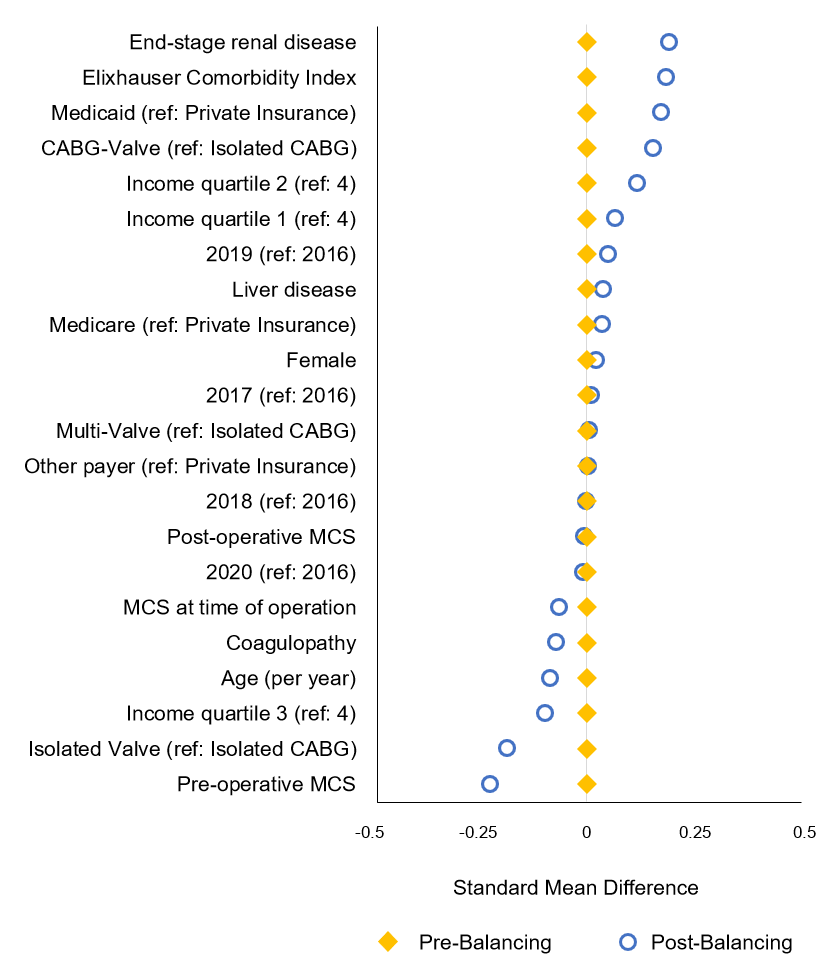

Supplement: S1 Figure — (TIF) [file pone.0319563.s001.tif]
